# Rates of tooth movement and bone remodeling activity: Self-ligating versus conventional brackets

**DOI:** 10.4317/jced.56615

**Published:** 2020-04-01

**Authors:** Leonard-Euler-Andrade-Gomes do Nascimento, Matheus-Melo Pithon, Antônio-Carlos de O. Ruellas, Eduardo-Sant`Anna Franzotti, Antônio-Cruz-Gonçalves Filho, Margareth-Maria-Gomes de Souza, Ana-Maria Bolognese

**Affiliations:** 1 PhD, Student at the Department of Orthodontics, School of Dentistry, Federal University of Rio de Janeiro - UFRJ, Rio de Janeiro, Brazil; 2 Professor of Orthodontics, Department of Health I, School of Dentistry, Southwest Bahia State University - UESB, Jequié, Bahia, Brazil; 3 Professor of Orthodontics Department of Pediatric Dentistry and Orthodontics, School of Dentistry, Federal University of Rio de Janeiro - UFRJ, Rio de Janeiro, Brazil; 4 Specialist in Orthodontics, Department of Orthodontics, School of Dentistry, Federal University of Piauí - UFPI, Teresina, Piauí, Brazil

## Abstract

**Background:**

Bracket systems have been developed with the purpose of reducing frictional resistance between the archwire and accessories. The aim of this research was to compare rates of tooth movement and regions of cellular bone modeling activity along tooth root surfaces of teeth moved with conventional vs. self-ligating brackets.

**Material and Methods:**

The experiments were conducted in 20 male dogs. Bands were cemented in all intermediate incisors, with conventional brackets (Morelli) on the right side and hybrid self-ligating ones (T3-American Orthodontics) on the left side. A 0.019” x 0.025” stainless steel wire was inserted passively in the slot of these brackets with chain elastics (250 gf) to perform sliding mechanics. Clinical records of the orthodontic mechanics were made before and after 15 days of the tooth movement. The dental segments of the animals were prepared for light microscopy. Statistical analysis of variance and the Tukey correction with a P value at 5% were used.

**Results:**

There were no significant differences in tooth movement rates between the two types of brackets but differences, in the bone modeling activity, suggested that tooth movement with the self-ligating brackets resulted in more tipping and less translational movement than tooth movement with the conventional brackets.

**Conclusions:**

The rates of tooth movement were similar between the two systems. The histological evaluation of cellular bone modeling activity along tooth root surfaces showed more translation movement of teeth with the conventional brackets, and more tipping movement of teeth with self-ligating brackets.

** Key words:**Edgewise, histological reactions, orthodontic movement, self-ligating, brackets.

## Introduction

Friction is particularly determined by the ligation method used, which can be elastomeric ligatures, wire ligatures or ligating clips (1). The self-ligating bracket systems have been developed with the purpose of reducing frictional resistance between the archwire and accessories. Some are considered: passive, with rigid clips (Damon, Smart Clip, Vision); active, with flexible clips that press against the archwire constantly irrespective of thickness (Speed, In-Ovation); and hybrid (passive and active) depending on the diameter and position of the orthodontic archwire (T3) (2-4).

In the literature (3,5-8) there are reports that once the bracket systems cause less amount of friction, they significantly reduce treatment time during sliding mechanics. The bracket systems, be the edgewise self-ligating (SL) or conventional edgewise (EW) bracket systems, should promote to the bracket/orthodontic wire system the lowest amount of friction possible, (7,9-11) but without impairing the quality of movement planned. The idealized SL brackets with different shapes, sizes, mechanics, and a considerable ability to reduce friction (12-15) are widely used in clinical routine.

A systematic review article (16) investigated the influence of SL bracket type on alignment efficiency, subjective pain experience, bond failure rate, arch dimensional changes, rate of orthodontic space closure, periodontal outcomes, and root resorption. This review outcomes (16) showed: a) insufficient evidence to support the use of SL fixed orthodontic appliances over EW appliance systems or vice versa, b) SL do not confer particular advantage with regard to subjective pain experience and, c) insufficient evidence suggesting that orthodontic treatment is more or less efficient with SL. There are reports that some SL bracket systems present less amount of friction, (6-15) allowing greater orthodontic movement, however, there is no information about the cellular bone modeling activity of the movement achieved. Therefore, the aim of this study was to assess the biomechanical behavior of the SL and EW brackets, observing the following responses: the rates of orthodontic movement observed through clinical evaluation, and its cellular bone modeling activity, through the initial histological reactions of the periodontal ligament (PDL) after the application of sliding mechanics.

## Material and Methods

The research was approved by the Ethics Committee for Animal Experimentation under report number 01/09. Throughout the entire experiment, the experimental procedures on the animals fulfilled the Proposed International Ethical Guidelines for Biomedical Research involving animals (Council for International Organizations of Medical Sciences – CIOMS/WHO, 1985). 20 male dogs of non-defined breed (NDB) adults of age 3 years (+/- 0.5 years) and mean weight of 12 kg (+/- 1 kg) were subjected to quarantine and recruited to participate in this study. For the sedation procedure of the animals, the following drugs were administered intramuscularly: 0.7ml of acepromazine (Acepran-0.1%-Univet), 0.8ml of ketamine hydrochloride (Vetanarcol-König) and 0.8ml of dihidro-tiazine hydrochloride (Rompum-Bayer).

The prophylaxis of the teeth was performed weekly and, for each animal, randomly selected lateral incisors served as the control units, which were not orthodontically treated and they were used as parameters for the observation of the clinical and histological aspects with regard to the normal biological development of the bone structures, teeth, and periodontal tissue. As part of the required orthodontic mechanics, the central incisors were extracted from the mandible and maxilla on both sides, then bands with EW brackets (Morelli, Sorocaba, São Paulo) with a 0.022” x 0.028” slot were bonded to the maxillary and mandibular intermediate incisors on the right side, and hybrid SL brackets (T3-American Orthodontics) with a 0.022” x 0.028” slot to the corresponding teeth on the left side. All brackets were of the same slot prescription and the bands were bonded with Transbond XT (3M Unitek). The same type of resin was added in the incisal portion of the bands in order to provide greater mechanical retention. The 0.019” x 0.025” stainless steel wires (CrNi) of the brand Unitek were passively inserted in the slots of all brackets. To tie the wire to the EW bracket slots, a 0.008” ligature wire was used.

**Figure 1:**
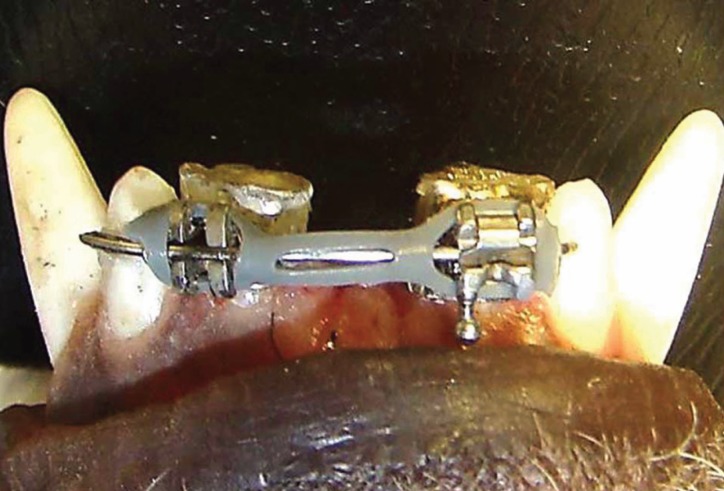
The 0.019” x 0.025” stainless steel wires (CrNi) passively inserted in the slots of all the brackets and after 30 days of the initial alignment of band slots, the sliding mechanics activation was begun using gray chain elastics (250gf), from the brackets of the intermediate incisors on the left side to the ones on the right side on the mandibular and maxillary dental arches.

After 30 days of the initial alignment of band slots, the sliding mechanics activation was performed using gray chain elastics (Morelli, Sorocaba, São Paulo) with a load of 250gf from the brackets of the intermediate lateral incisors on the left side to the ones on the right side on the mandibular and maxillary dental arches (Fig. 1).

-Assessment of the rates of orthodontic movement

An electronic digital caliper (0-100 mm) was used to record the rates of orthodontic movement on each hemi-arch of the mouth of the animals, using the following references: 1) the mesial surface, close to the gingival third of the canines crown, were perforated with a spherical diamond bur No 1013 (KG) and 2) the distal surface of the intermediate incisor bracket at the level of the cervical winglet. The mean value of the three consecutive distance measurements, in millimeters, of the four hemi-arches was recorded by the same operator, who was previously calibrated. These recordings were performed at the beginning of orthodontic mechanics, just before the activation with gray chain elastics (T0), and after 15 days (T15). After this period, all the animals were euthanized with a lethal dose of anesthetic infused through the external carotid artery.

-Assessment of the cellular bone modeling activity

The dentoalveolar segments of interest were identified, dissected, placed in 4% paraformaldehyde and phosphate buffer (0.1 M PBS), and prepared for light microscopy analysis. The slices were cut in the vertical direction following the longitudinal axis of the roots. The slices were submitted to staining by the Harris hematoxylin-eosin method (Merck) and then mounted with Entellan (Merck). The reading of the histological structures was performed with the aid of the HM-LUX Nikon E600 microscope under the following resolution: 4NF x 0.10 . A system with computerized image analysis (Qwin Leica D-1000, version 4.1) captured 60 fields per tooth, 30 evenly distributed on the medial and distal sides, among the gingival, middle and apical thirds of the teeth controls as well as those moved with EW and SL brackets. The bone histomorphometry (17) performed the assessment of the bone modeling activity and estimated quantitatively the osteoclasts and osteoblasts in the 60 histological captured fields.

-Statistical Analysis

The data encountered was organized in Tables and box/whisker plots. Statistical analysis was performed to compare the right side and left side incisors tooth movement, using a paired non-parametrical test. The one-way ANOVA assumed the 4 sites: right (EW) and left (SL) * maxilla and mandible (2*2=4), which were independent with each other. To the mean difference between brackets and jaws, a descriptive statistics by the analysis of variance (ANOVA) and multiple comparisons with the Tukey’s test (*p*-value=5% of probability) were used.

## Results

-Assessment of the rates of orthodontic movement

**Figure 2:**
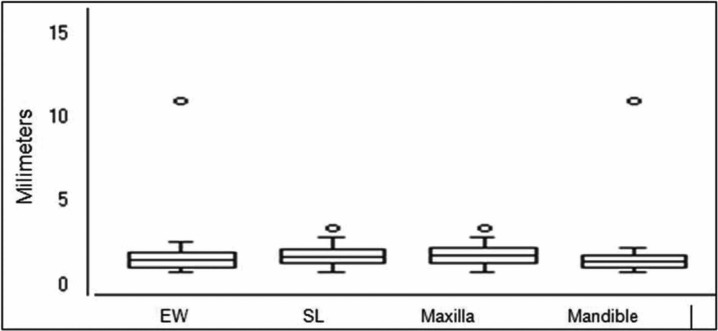
Differences between the rate of tooth movement (T15-T0), caused by the use of self-ligating brackets compared quantitatively to that caused by conventional brackets, after application of sliding mechanics. Width of each box represents the four groups assessed (EW, SL, Maxilla and Mandible). Box height represents differences in the first (25%) and third (75%) quartiles. Middle band in each box represents the median. Whiskers represent the minimum and maximum differences within 1.5 times the interquartile range of the lower and upper quartiles. Dots represent T15-T0 exceeding 1.5 times the interquartile range.

Table 1 and Figure 2 show that the rates of orthodontic movement had equal clinical significance between the conventional and self-ligating edgewise brackets systems when they were evaluated on the same bone arch, i.e., either the mandible or maxilla, as well as when evaluating the mandible and maxilla with the same system used (either EW or SL), a statistically significant difference was not found for the EW and SL brackets, which showed the same clinical movement behavior on both bone arches.

**Table 1:**
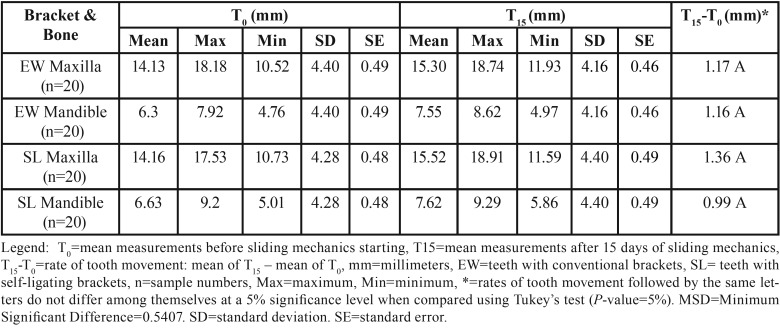
Mean measurements in millimeters(mm) noting the rate of tooth movement (T15-T0), caused by the use of self-ligating brackets compared quantitatively to that caused by conventional brackets, after application of sliding mechanics.

-Assessment of the cellular bone modeling activity

**Figure 3:**
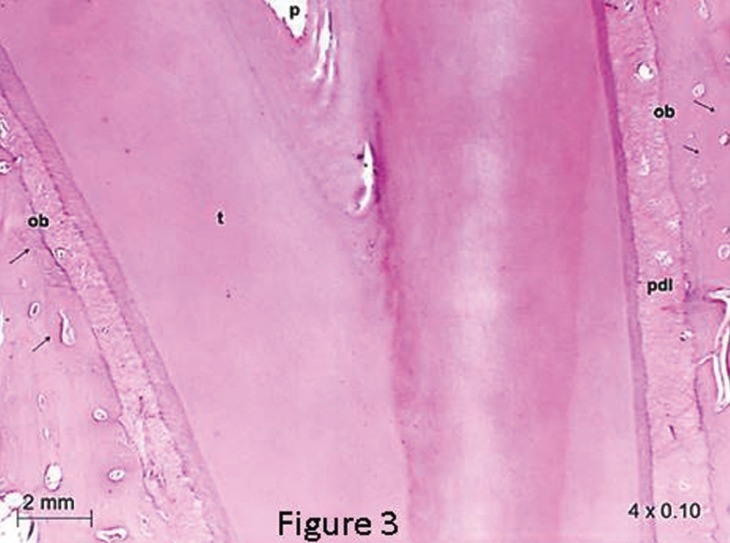
Photomicrograph of the control (C) lower left lateral incisor, at magnification of 4x0.10, shows aspects of normality for the PDL. p=pulp; pdl=periodontal ligament; t=root tooth; b=bone; ob=osteoblasts; arrows=incremental lines growth.

When evaluating the control teeth, the periodontal ligament (PDL) showed regular and uniform thickness throughout entire root (Fig. 3). In general, the collagen fibers remained parallel among them and they were perpendicularly inserted into the bone and cementum surfaces. Most fibroblasts presented a fusiform shape and were arranged in fascicles, whereas inflammatory cells were rarely found. A uniform distribution of blood vessels and nerves of various sizes were found throughout the periodontal ligament. The cementum surface was uniform and continuous. The bone crest was slightly irregular with the presence of a few osteoclasts (Table 2) arranged in Howship’s lacunae or juxtaposed to the bone surface. The osteoblasts were juxtaposed in the control teeth, but the osteoblasts were arranged with no organization in the teeth with both EW and SL brackets on the side of tension along the bone surfaces.

**Table 2:**
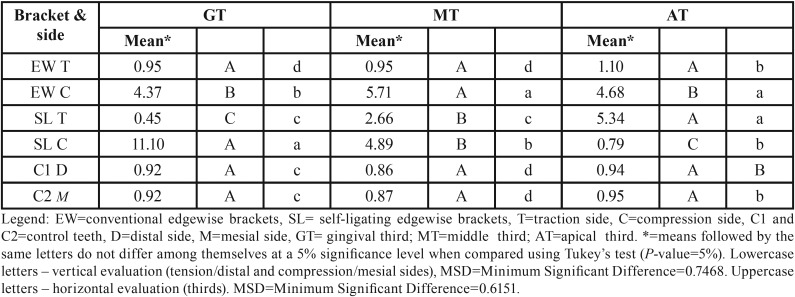
The cell count of osteoclasts performed on the sides of tension and compression, at the level of the gingival, middle and apical thirds (teeth with SL and EW systems), and mesial and distal sides of the control group teeth.

**Figure 4:**
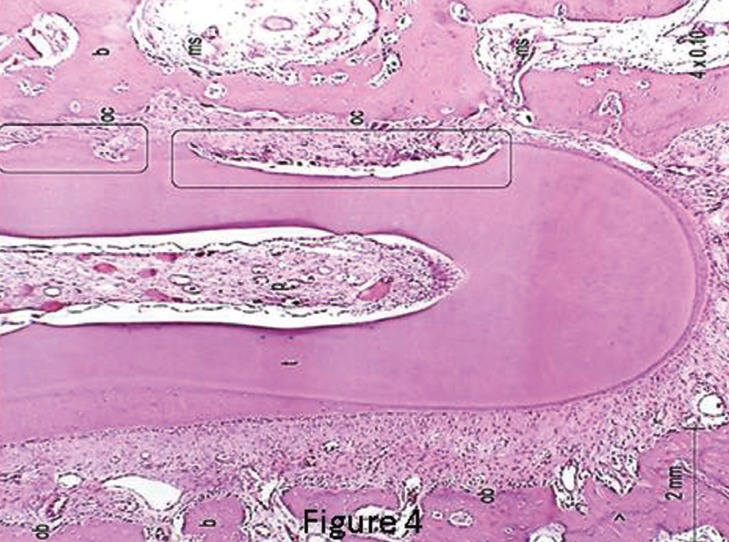
Panoramic view (4x0.40) of the cross-section at the middle third of the right intermediate incisor root (EW) shows, on the side of compression, osteoclasts (oc), root (squares) and bone absorption (frontal and undermining), and on the side of tension, osteoblasts arranged on the bone edge (ob), apposition of cementum and incremental lines growth (arrows); p=pulp; me=marrow space; t=root tooth; b= bone.

On the side of tension in the periodontium of the teeth with EW brackets, we observed intense osteoblast activity arranged from the cervical to the apical thirds of the bone edges, and the formation of cementum, indicating the presence of incremental growth lines (Fig. 4). In the periodontium of these teeth, adjacent to the bone surface at the level of the gingival, middle and apical thirds, irregular bone with active osteoclasts, located within Howship’s lacunae, was frequently found on the mesial surface (Table 2), which corresponded to the side that received compression. Loss of organization and individuality of Sharpey’s fibers, showing areas of frontal and undermining bone absorption, were observed (Fig. 4). The reactions of apposition (on the side of tension) and absorption (on the side of compression) were frequent along the roots of teeth with the EW brackets, suggesting translational movement of the teeth because in the osteoclasts and osteoblasts count, the amount of these cells were uniformly distributed along the sides with compression and tension, respectively, what can be seen in  Table 2 and  Table 3, showing the same statistical significance at the assessed root thirds.

**Table 3:**
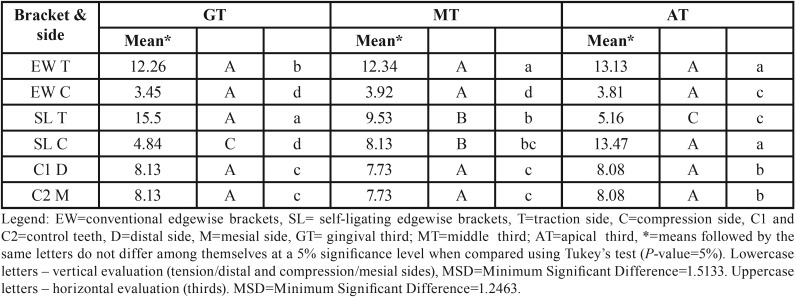
The cell count of osteoblasts performed on the sides of tension and compression, at the level of the gingival, middle and apical thirds (teeth with SL and EW systems), and mesial and distal sides of the control group teeth.

**Figure 5:**
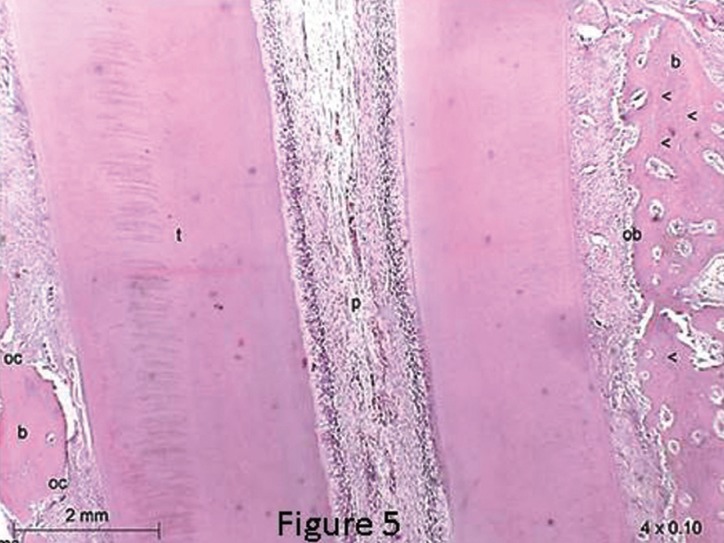
Photomicrographs of intermediate lower left incisor (SL) shows: irregular bone, significant number of active osteoclasts with frontal and undermining absorption at the gingival third of the root on the side of compression and the marginal bone crest with incremental lines growth and osteoblasts in the bone edge. Magnification of 4x0.10. b=bone; t=root tooth; pdl=periodontal ligament; ob=osteoblasts; oc=osteoclasts; me=marrow space; p=polp; arrows=incremental lines growth.

**Figure 6:**
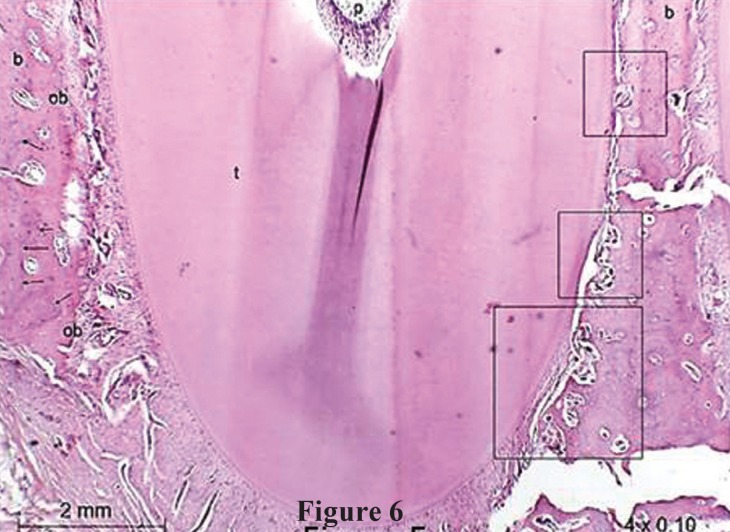
Photomicrographs of intermediate upper left incisor (SL) shows: irregular bone, significant number of active osteoclasts (square) with frontal and undermining absorption at the apical third of the root on the side of tension and incremental lines growth and osteoblasts in the bone edge at the compression side. Magnification of 4x0.10. b=bone; t=root tooth; pdl=periodontal ligament; ob=osteoblasts; oc=osteoclasts; me=marrow space; p=polp; arrows=incremental lines growth.

In teeth that have been moved with SL brackets, areas of frequent absorption were found with irregular bone, significant number of active osteoclasts in gingival thirds on the side of compression (Fig. 5) and in the apical region of the roots on the side of tension (Fig. 6). On the side of tension at the apical third was also found teeth with areas of root absorption and frontal and undermining bone absorption, with predominance of the latter. The osteoblasts count on the side of tension, at the gingival third, showed equal statistical significance in comparison with the side with compression at the apical third (Table 3). The same statistical significance was also observed in the osteoclasts count, when observing both the compression side at the gingival third and the tension side at the apical third (Table 2). This indicates similar tissue reactions in these two areas, despite being on different sides and thirds, which findings suggest tipping movement of the teeth that received the SL brackets.

## Discussion

In the present study, we investigated the rates of orthodontic tooth movement and cellular bone modeling activity within 15 days, among conventional brackets that included wire ligatures and self-ligating brackets that did not use ligatures. The rates of the orthodontic movement with sliding mechanics were observed through clinical evaluation, and its cellular bone modeling activity, due to the responses of periodontal ligament cells (osteoclastos and osteoblasts). A verification of slot alignment and efforts to eliminate the friction or resistance generated due to differences in initial alignment of band slots was done to enhance the validity of our findings. So, the 0.019” x 0.025” stainless steel wires (CrNi) were passively inserted in the slots of all brackets, and just after 30 days of the initial alignment of band slots, the sliding mechanics activation had been begun.

Good reasons support the use of dogs as experimental animals in induced tooth movement. It has been demonstrated that transformation of bone structures in animals is basically the same for humans, what is seen by radiographic and histological examination of these two models (18). We performed the present study to investigate the rates of tooth movement and bone modeling activity considering a short follow up time with animals models, because it is totally comparable with those ones proceeded in humans (19).

Our research used the bone histomorphometry because it enables the quantitative assessment of bone turnover and remodeling in histological sections of bone (17). It provides unique information about mechanisms of bone loss and gain in therapeutic interventions (17). Many of the measurements made in bone histomorphometry have a subjective element, and this, together with differences in staining techniques, magnification, and measurement methods, contributes to significant inter- and intra-observer measurement variability (20,21).

The application of force of 250 gf within 15 days was sufficient to observe slight clinical movement and significant histological reactions. Approximately two days after the application of orthodontic force, local modifications allow the osteoblasts and osteoclasts to begin the process of bone remodeling, with apposition on the side on which there is tension and absorption on the same on which there is compression (23-28). These histological reactions were also observed in our study, but with greater presence of absorption areas than of apposition areas. In this research, the performance of clinical movement in the time interval of fifteen days did not show statistical significance between the EW and SL brackets, which is in agreement with the study (15) conducted with the passive Smart Clip SL brackets and EW brackets in the same way as those of the studies (29,30) with Damon 2 and EW brackets. However, a different performance of the initial histological reactions of bone absorption and apposition was found between the EW and SL brackets. In the periodontal ligament of the teeth with EW brackets, an agglomeration of osteoblasts and osteoclasts were uniform along the root thirds of the teeth of these animals, while for the SL brackets, the prevalence of these cells was more at the gingival or apical third. In our study, on the side of tension in the teeth with SL brackets, compression was found at the apical third and tension at the gingival third. Instead, on the side of compression, the opposite occurred, suggesting that the tipping movement was present in the teeth with SL brackets. The histologically identified tipping movement may have influenced the outcome of the quantification of clinical movement, which was slightly higher in the maxillary teeth with these brackets, however with no statistical significance. In the teeth groups with EW, tension and compression were arranged along the root thirds of the teeth only on the corresponding sides, suggesting translational movement for this group.

The degree of success achieved with orthodontic mechanics can be influenced by many variables such as: age, endocrine factors, systemic diseases, medication, bone density, type of material, size, shape and angle of the wire interface/slot, humidity and bond strength and ligation form (31-35). In our study, when evaluating the orthodontic behavior between the mandible and maxilla arches using the same system (either EW or SL), a statistically significant difference was not found for the brackets between the two bone arches, even the values at the mandible arch had showed the lowest rate of tooth movement. The bone responds differently to orthodontic force: clinically, maxillary and mandibular, resulting in a signiﬁcant difference in the rates of tooth movement (36). Another study say that in the skeletally mature (1- to 2-year-old) dogs, the bone formation rate (BFR), which is a measure of the bone turnover or remodeling in bone supporting permanent teeth, is substantially higher in the alveolar process surrounding erupted permanent teeth (mandible approximately 37%/y, maxilla approximately 19%/y) than the BFR found at other sites such as the femur (6.4%/y) (37).

Our outcomes showed that the SL brackets had the same clinical movement behavior on both bone arches, however, they suffered greater tipping movement than translational movement, which is in agreement with some authors (38-40) affirming that there is sufficient evidence that large rectangular wires, in the presence of tipping, the SL brackets can cause less friction in comparison with the EW brackets and the performance of orthodontic mechanics of EW and SL brackets may be related to the size of the orthodontic wire and not related only to the system used. However, how others studies (41) reported the application of methods in modern molecular biology focused on the role played by the periodontal ligament cells, further researches can explain which system could anticipate cellular stress even before starting orthodontic movement.

## Conclusions

The conventional edgewise and self-ligating systems showed similar performance in the clinical rates of the orthodontic movement, however, the initial histological reactions of cellular bone modeling activity, along tooth root surfaces of teeth moved with conventional vs. self-ligating brackets, indicated translation movement for conventional edgewise orthodontic brackets and tipping movement in teeth with self-ligating brackets.

## References

[1] Raveli DB, Goes DR, Dib LPS, Oyonarte R (2008). Self-ligating brackets system: the great tendency on contemporary orthodontics. Rev Clin Ortodon Dental Press.

[2] Agarwal S, Valiathan A, Shah NV (2008). Self-ligating brackets. Am J Orthod Dentofacial Orthop.

[3] Cacciafesta V, Sfondrini MF, Scribante A, Klersy C, Aurichio F (2003). Evaluation of friction of stainless steel and esthetic self-ligating brackets in various bracket-archwire combinations. Am J Orthod Dentofacial Orthop.

[4] Rinchuse DJ, Miles PG (2007). Self-ligating brackets: present and future. Am J Orthod Dentofacial Orthop.

[5] Pandis N, Strigou S, Eliades T (2006). Maxillary incisor torque with conventional and self-ligating brackets: a prospective clinical trial. Orthod Craniofac Res.

[6] Damon DH (1998). The rationale, evolution and clinical application of the self-ligating bracket. Clin Orthod Res.

[7] Fernandes DJ, Almeida RCC, Quintão CCA, Elias CN, Miguel JAM (2008). The self-ligating system in an aesthetic view. R Dental Press Ortodon Ortop Facial.

[8] Yu YL, Qian YF (2007). The clinical implication of self-ligating brackets. Shanghai Kou Qiang Yi Xue.

[9] Scott P, Sherriff M, Dibiase AT, Cobourne MT (2008). Perception of discomfort during initial orthodontic tooth alignment using a self-ligating or conventional bracket system: a randomized clinical trial. Eur J Orthod.

[10] Berger JL (1990). The influence of the SPEED bracket's self-ligating design on force levels in tooth movement: a comparative in vitro study. Am J Orthod Dentofacial Orthop.

[11] Pizzoni L, Ravnholt G, Melsen B (1998). Frictional forces related to self-ligating brackets. Eur J Orthod.

[12] Badawi HM, Toogood RW, Carey JP, Heo G, Major PW (2008). Torque expression of self-ligating brackets. Am J Orthod Dentofacial Orthop.

[13] Henao SP, Kusy RP (2004). Evaluation of the frictional resistance of conventional and self-ligating bracket designs using standardized archwires and dental typodonts. Angle Orthod.

[14] Loftus BP, Artun J, Nicholls JI, Alonzo TA, Stoner JA (1999). Evaluation of friction during sliding tooth movement in various bracket-arch wire combinations. Am J Orthod Dentofacial Orthop.

[15] Miles PG (2007). Self-ligating vs conventional twin brackets during en-masse space closure with sliding mechanics. Am J Orthod Dentofacial Orthop.

[16] Fleming PS, Johal A (2010). Self-ligating brackets in orthodontics. A Systematic review. Angle Orthod.

[17] Compston J (2004). Bone Histomorphometry -- The Renaissance? BoneKEy-Osteovision. http://www.bonekey-ibms.org/cgi/content/full/ibmske;1/5/9..

[18] Reitan K, Kvam E (1971). Comparative behavior of human and tissue during experimental tooth movement. Angle Orthod.

[19] Graber TM, Vanarsdall RL (1996). Princípios e técnicas atuais. 2. ed.

[20] de Vernejoul MC, Kuntz D, Miravet L, Goutallier D, Ryckewaert A (1981). Bone histomorphometric reproducibility in normal patients. Calcif Tissue Int.

[21] Chavassieux PM, Arlot ME, Meunier PJ (1985). Intermethod variation in bone histomorphometry: comparison between manual and computerized methods applied to iliac bone biopsies. Bone.

[22] Wright CD, Vedi S, Garrahan NJ, Stanton M, Duffy SW, Compston JE (1992). Combined inter-observer and intermethod variation in bone histomorphometry. Bone.

[23] Consolaro A (2002). Movimentação dentária induzida: biologia aplicada à prática clínica. In: Consolaro A. Reabsorções Dentárias nas Especialidades Clínicas.

[24] Roberts WE, Huja S, Roberts JA (2004). Bone modeling: biomechanics, molecular mechanisms, and clinical perspectives. Sem Orthod.

[25] Melsen B (1999). Biological reaction of alveolar bone to orthodontic tooth movement. Angle Orthod.

[26] Sutherland MK, Geoghegan JC, Yu C, Turcott E, Skonier JE, Winkler DG (2004). Sclerostin promotes the apoptosis of human osteoblastic cells: a novel regulation of bone formation. Bone.

[27] Mackie EJ (2003). Osteoblasts: novel roles in orchestration of skeletal architecture. Int J Biochem Cell Biol.

[28] Sandy JR, Farndale RW, Meikle MC (1993). Recent advances in understanding mechanically induced bone remodeling and their relevance to orthodontic theory and practice. Am J Orthod Dentofacial Orthop.

[29] Pandis N, Polychronopoulou A, Eliades T (2007). Self-ligating vs conventional brackets in the treatment of mandibular crowding: a prospective clinical trial of treatment duration and dental effects. Am J Orthod Dentofacial Orthop.

[30] Miles PG, Weyant RJ, Rustveld L (2006). A Clinical Trial of Damon 2Y Vs Conventional Twin Brackets during initial alignment. Angle Orthod.

[31] Henao SP, Kusy RP (2005). Frictional evaluations of dental typodont models using four self-ligating designs and a conventional design. Angle Orthod.

[32] Pandis N, Eliades T, Partowi S, Bourauel C (2008). Forces exerted by conventional and self-ligating brackets during simulated first- and second-order corrections. Am J Orthod Dentofacial Orthop.

[33] Kim TK, Kim KD, Baek SH (2008). Comparison of frictional forces during the initial leveling stage in various combinations of self-ligating brackets and archwires with a custom-designed typodont system. Am J Orthod Dentofacial Orthop.

[34] Zachrisson BU (2006). Use of self-ligating brackets, superelastic wires, expansion/proclination, and permanent retention - a word of caution. World J Orthod.

[35] Turnbull NR, Birnie DJ (2007). Treatment efficiency of conventional vs self-ligating brackets: effects of archwire size and material. Am J Orthod Dentofacial Orthop.

[36] Deguchi T, Takano-Yamamoto T, Yabuuchi T, Ando R, Roberts WE, Garetto LP (2008). Histomorphometric evaluation of alveolar bone turnover between the maxilla and the mandible during experimental tooth movement in dogs. Am J Orthod Dentofacial Orthop.

[37] Huja SS, Fernandez SA, Hill KJ, Li Y (2006). Remodeling dynamics in the alveolar process in skeletally mature dogs. Anat Rec A Discov Mol Cell Evol Biol.

[38] Ehsani S, Mandichb MA, El-Bialy TH, Flores-Mir C (2009). Frictional resistance in self-ligating orthodontic brackets and conventionally ligated brackets A Systematic Review. Angle Orthod.

[39] Morina E, Eliades T, Pandis N, Jäger A, Bourauel C (2008). Torque expression of self-ligating brackets compared with conventional metallic, ceramic, and plastic brackets. Eur J Orthod.

[40] Harradine NW (2001). Self-ligating brackets and treatment efficiency. Clin Orthod Res.

[41] Kumamoto H, Yamauchi K, Yoshida M, Ooya K (2003). Immunohistochemical detection of matrix metalloproteinases (MMPs) and tissue inhibitors of metalloproteinases (TIMPs) in ameloblastomas. J Oral Pathol Med.

